# Pharmacological suppression of the kallikrein kinin system with KVD900: An orally available plasma kallikrein inhibitor for the on‐demand treatment of hereditary angioedema

**DOI:** 10.1111/cea.14122

**Published:** 2022-03-20

**Authors:** Edward J. Duckworth, Nivetha Murugesan, Lily Li, Louise J. Rushbrooke, Daniel K. Lee, Gian Marco De Donatis, Andreas Maetzel, Christopher M. Yea, Sally L. Hampton, Edward P. Feener

**Affiliations:** ^1^ 319952 KalVista Pharmaceuticals Cambridge Massachusetts USA; ^2^ Institute for Health Policy, Management & Evaluation University of Toronto Toronto Ontario Canada

**Keywords:** hereditary angioedema, kallikrein kinin system, plasma kallikrein inhibitor

## Abstract

**Background:**

Hereditary angioedema (HAE) is a rare genetic disease that leads to recurrent episodes of swelling and pain caused by uncontrolled plasma kallikrein (PKa) activity. Current guidelines recommend ready availability of on‐demand HAE treatments that can be administered early upon attack onset. This report describes the pharmacological and pharmacodynamic properties of the novel oral small‐molecule PKa inhibitor KVD900 as a potential on‐demand treatment for HAE.

**Methods:**

Pharmacological properties of KVD900 on PKa and closely related serine proteases were characterized using kinetic fluorogenic substrate activity assays. Effects of KVD900 on PKa activity and kallikrein kinin system activation in whole plasma were measured in the presence of dextran sulphate (DXS)‐stimulation using a fluorogenic substrate and capillary immunoassays to quantify high molecular weight kininogen (HK), plasma prekallikrein and Factor XII cleavage. Pharmacodynamic effects of orally administered KVD900 were characterized in plasma samples from six healthy controls in a first in human phase 1 clinical trial and from 12 participants with HAE in a phase 2 clinical trial.

**Results:**

KVD900 is a selective, competitive and reversible inhibitor of human PKa enzyme with a K_i_ of 3.02 nM. The association constant (K_on_) of KVD900 for PKa is >10 × 10^6^ M^−1^ s^−1^. Oral administration of KVD900 in a first‐in‐human clinical trial achieved rapid and near complete inhibition of DXS‐stimulated PKa enzyme activity and HK cleavage and reduced plasma prekallikrein and Factor XII activation in plasma. In individuals with HAE, orally administered KVD900 inhibited DXS‐stimulated PKa activity in plasma by ≥95% from 45 min to at least 4 h post‐dose and provided rapid protection of HK from cleavage.

**Conclusion:**

KVD900 is a fast‐acting oral PKa inhibitor that rapidly inhibits PKa activity, kallikrein kinin system activation and HK cleavage in plasma. On‐demand administration of KVD900 may provide an opportunity to halt the generation of bradykinin and reverse HAE attacks.

**Key Message**

- KVD900 is a novel, oral and selective plasma kallikrein (PKa) inhibitor with high potency in plasma and rapid absorption.
- Orally administered KVD900 provides rapid and near complete inhibition of PKa and KKS activation in both control and HAE participants.
- On‐demand administration of KVD900 may provide an opportunity to halt the generation of PKa and bradykinin and reverse HAE attacks.

## 1 INTRODUCTION

Hereditary angioedema (HAE) is a rare genetic disease that causes recurrent episodes of angioedema of skin and mucosal membranes.^1^, ^2^ Attacks of angioedema are commonly painful, debilitating and can become life‐threatening when affecting the upper airways.^3^ The frequency, severity and location of attacks are highly variable.^4^ Molecular and pharmacological studies have demonstrated that spontaneous uncontrolled plasma kallikrein (PKa) enzyme activity is a primary cause of HAE attacks.^5^, ^6^

Most known mutations associated with HAE facilitate the actions of the PKa‐kinin system (KKS).^7^ The most prevalent and well‐characterized causes of HAE involve mutations in the *SERPING1* gene, which result in either reduced expression (HAE type 1) or function (HAE type 2) of C1‐inhibitor (C1‐INH).^8^ C1‐INH is a serine protease inhibitor (in the serpin family) that circulates abundantly in the blood and covalently binds and inactivates both PKa and Factor XIIa (FXIIa). These serine proteases of the KKS are derived from their respective zymogens, plasma prekallikrein (PK) and FXII. Reciprocal activation of these zymogen by FXIIa and PKa is accelerated upon the interactions of the KKS with negatively charged natural and artificial surfaces, which is also referred to as contact system activation.^9^ PKa activity thereby contributes to the formation of FXIIa from FXII and cleaves the PKa substrate, high molecular weight kininogen (HK) resulting in the generation of the hormone bradykinin (BK).^9^ BK and its metabolite desArg^9^ BK activate the cell surface receptors B2R and B1R, respectively, resulting in increased vascular permeability and pro‐inflammatory responses.^10^, ^11^ C1‐INH deficiency facilitates uncontrolled PKa‐mediated generation of BK and angioedema during an HAE attack.^12^

Currently approved treatments for HAE include C1‐INH (recombinant and plasma‐derived), PKa inhibitors (lanadelumab, ecallantide and berotralstat), and the B2R antagonist icatibant.^13^ C1‐INH, ecallantide and icatibant are approved as on‐demand treatments administered by injection upon onset of symptoms. Current HAE treatment guidelines recommend rapid access to an effective on‐demand medication to administer at the onset of all attacks for all patients with HAE.^14^, ^15^ Early administration of on‐demand medications has been shown to shorten attack duration, reduce severity and provide faster symptom relief.^16^, ^17^ An orally administered and fast acting on‐demand treatment for HAE may provide patients the opportunity to treat attacks as soon as possible after onset, consistent with treatment guidelines and thereby halt attack progression.

We are developing KVD900 as an orally available, potent and selective PKa inhibitor for the on‐demand treatment of HAE attacks. This paper characterizes the pharmacological properties of KVD900 and its pharmacodynamic effects on PKa activity and KKS activation in plasma following oral administration in healthy controls and in individuals with HAE.

## 2 METHODS

### 2.1 Pharmacology of KVD900 in isolated enzyme and whole plasma PKa assays

#### 2.1.1 PKa affinity (Ki) and modality of binding

The inhibitory effects of KVD900, following pre‐incubation for 5 min, on the enzymatic activity of PKa were analysed with kinetic assays of fluorogenic substrate cleavage using a fluorometer (Spark 20M, Tecan). PKa (Calbiochem) activity was measured using H‐D‐Pro‐Phe‐Arg‐AFC (Peptide Protein Research), at concentrations ranging from 0.1‐ to 10‐fold of the K_m_. To estimate *K_i_* and determine modality of binding, a mixed model of enzyme inhibition was fitted to the rate of fluorescence generated over a range of KVD900 concentrations using GraphPad Prism (GraphPad Software).

#### 2.1.2 Related protease IC_50_ determination

The effects of KVD900 on the catalytic activity of a panel of serine proteases (Table 1) were analysed using fluorogenic substrate cleavage assays specific for each protease. Protease inhibition was measured by monitoring the rate of fluorescence generation at a fixed substrate concentration over a range of inhibitor concentrations. KVD900 was pre‐incubated with the enzymes for 5 min prior to substrate addition. To estimate the IC_50,_ a 4‐parameter logistic dose‐response curve was fitted to the normalized rate of fluorescence increase.

**TABLE 1:** KVD900 protease selectivity

Enzyme	IC_50_
Plasma kallikrein	6.0 nM
Tissue kallikrein	>10 µM
FXIIa	>40 µM
FXIa	>40 µM
FXa	>10 µM
FVIIa	>10 µM
Plasmin	>40 µM
Thrombin	>40 µM
Trypsin	>40 µM
Beta‐secretase 1	>10 µM
Cathepsin D	>10 µM
Cathepsin G	>10 µM
Renin	>10 µM
Tissue plasminogen activator	>10 µM
Tryptase	>10 µM

Note

IC_50_ values for KVD900 inhibition of PKa and related proteases in isolated enzyme kinetic fluorogenic substrate assays.

#### 2.1.3 K_on_ determination

The association rate constant (K_on_) was determined by rapidly mixing inhibitor, over a range of 8 concentrations, with PKa enzyme and substrate (at K_m_). For each inhibitor concentration, the apparent association constant (K_obs_) was calculated as the half‐life of the decrease (first rate exponential decay) in free enzyme species as the inhibitor binds to the enzyme. K_on_ for a given compound was estimated by using the formula K_obs_ = K_off_ + K_on_*[I].

#### 2.1.4 PKa enzyme activity and protein binding in whole plasma

PKa enzyme activity was measured in human pooled plasma (control plasma, Affinity Biologicals) using H‐D‐Pro‐Phe‐Arg‐AFC. The KKS in plasma was stimulated by the addition of dextran sulphate 500 kDa (DXS; Sigma‐Aldrich). PKa activity in the plasma was estimated based on the maximum rate of fluorescence increase. To determine the PKa plasma IC_50_, compounds (at 8 concentrations) were pre‐incubated for 5 min in control plasma prior to DXS‐stimulation. Plasma samples were stimulated with 6.25 or 10 µg/ml DXS and PKa enzyme activity was measured as described above. Percent plasma protein binding (PPB %) was determined using Rapid Equilibrium Dialysis (Pierce RED technology, ThermoFisher Scientific). Compounds were spiked into control plasma (final concentration 5 μM) and dialysed against phosphate buffer (pH 7.4) for 5 h at 37°C. Samples were analysed using liquid chromatography‐tandem mass spectrometry to quantify the fraction of unbound compound.

### 2.2 Capillary‐based immunoassay of KKS components

Dose‐response experiments were performed on control plasma pre‐treated with KVD900 for 15 min followed by stimulation with 6.25 µg/ml DXS on ice for 17 min to induce KKS activation and PKa‐mediated HK cleavage. Undiluted plasma samples obtained at pre‐dose and post‐dose from healthy controls and HAE patients from clinical trials with KVD900, described below, were stimulated with DXS, as described above. Reactions were stopped by rapidly heating samples to 95°C for 10 min in Laemmli buffer containing β‐mercaptoethanol. Components of the KKS in plasma samples (diluted 1:20) were separated by capillary electrophoresis using 12–230 kDa separation modules in the Protein Simple WES^TM^ system (ProteinSimple). Proteins were detected by immunoassay and quantified from peak areas corresponding to chemiluminescence intensities using Compass for Simple Western software (Version 4.0.0, ProteinSimple) and visualized as virtual blots generated from capillary electropherograms as previously described.^18^ Standard curves were generated to estimate protein concentrations in plasma using known quantities of purified HK, plasma prekallikrein (PK), FXII and FXIIa (Enzyme Research Laboratories), spiked into HK (Affinity Biologicals), PK (Affinity Biologicals) and FXII (Technoclone) deficient plasma respectively. Immunodetection was performed using primary antibodies for PK (clone 13G11) (Invitrogen), HK and cHK: KNG17A12 Antibody (Molecular Innovations), FXII and FXIIa: FXII20B2 antibody (Molecular Innovations) and horseradish peroxidase‐conjugated secondary antibody. The anti‐HK light chain antibody used detected HK and two cHK bands. The total cHK was quantified by taking the sum of the peak areas for both cHK bands.

### 2.3 Clinical trial samples

Plasma samples from six healthy adult male volunteers were obtained at pre‐dose and up to 12 h post oral administration of 600 mg KVD900 formulated as powder in capsule in a phase 1 single‐ascending dose double‐blind, placebo‐controlled trial. Plasma samples from 42 adult male or female participants with a confirmed diagnosis of HAE type 1 or type 2 and at least 3 documented HAE attacks in the past 93 days were obtained at pre‐dose and up to 4 h after oral dosing with 600 mg KVD900 formulated in tablets, in the open label part of a phase 2 trial. Participants with HAE were within their intercritical period. Blood samples were collected into 3.2% sodium citrate, centrifuged at 1500 *g* for 10 min at 4°C, and plasma was stored at −80°C until analysis. Additional information on these trials can be found elsewhere (NCT04349800 and NCT04208412, available at https://clinicaltrials.gov).

### 2.4 Data analysis and statistics

Data were analysed in GraphPad Prism. One‐way analysis of variance (ANOVA) with Dunnett's multiple comparison test for significance was used to estimate statistical significance of differences between groups. *p*‐values <.05 were considered statistically significant. Pearson's correlation coefficient was calculated to estimate the strength of a linear association between 2 variables.

### 2.5 Ethical considerations

The Phase 1 trial protocol and subsequent amendments were approved by Wales Research Ethics Committee I, Cardiff, UK, and clinical trial authorization was obtained from the UK Medicines and Healthcare Products Regulatory Agency. The Phase 2 trial protocol and subsequent amendments were approved by the relevant Independent Ethics Committee (IEC)/Institutional Review Board (IRB)/Regulatory Authority in each of the participating countries. All participants provided written informed consent prior to initiation of study procedures. The trials were conducted according to global and local standards of Good Clinical Practice and in accordance with the Declaration of Helsinki (Brazil 2013).

## 3 RESULTS

### 3.1 KVD900 is a potent and selective plasma kallikrein inhibitor

**FIGURE 1:**
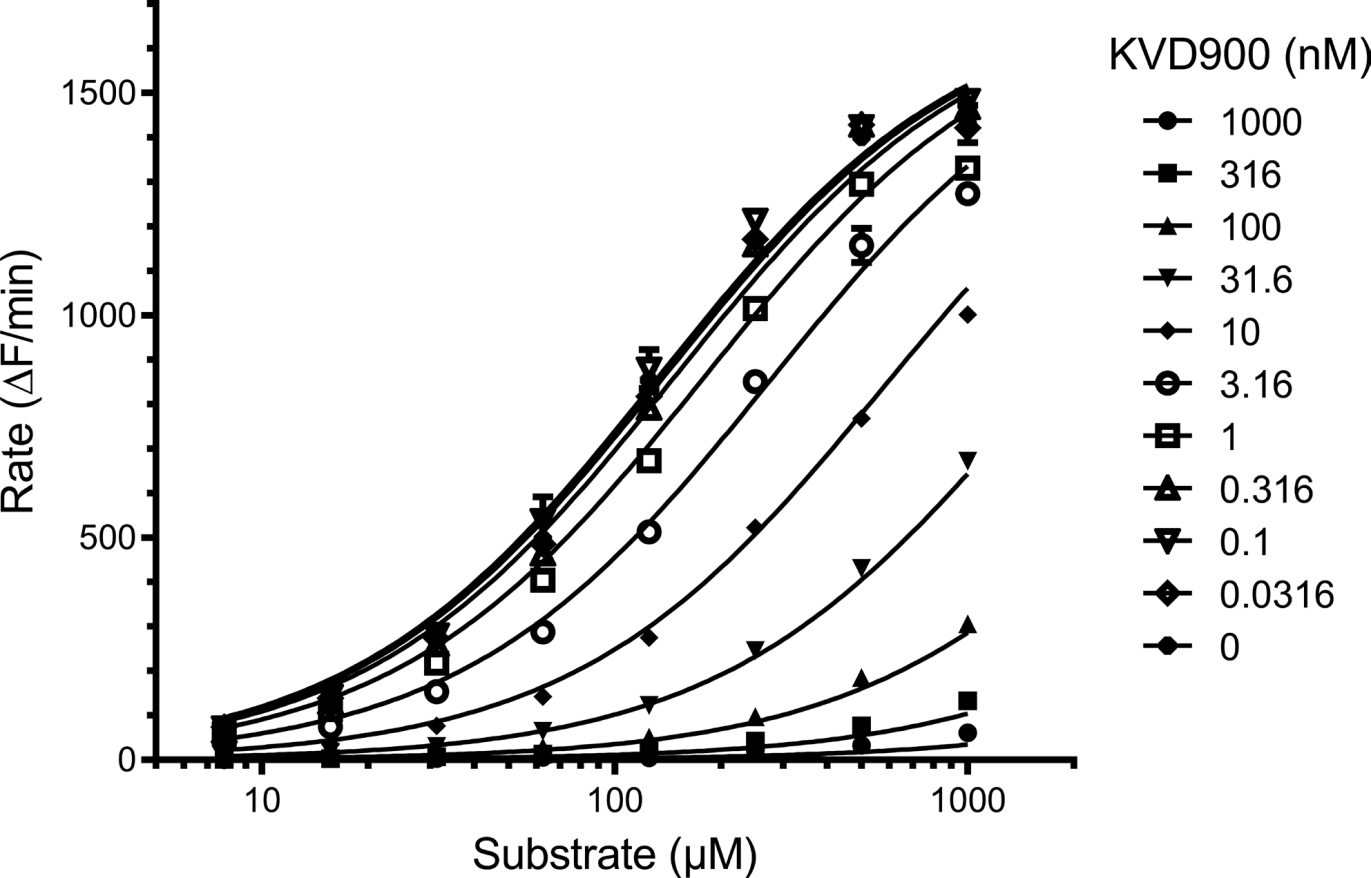
Effect of KVD900 in an isolated PKa enzyme K_i_ assay activity. Various concentrations of KVD900 were incubated with PKa for 5 min before the addition of substrate H‐D‐Pro‐Phe‐Arg‐AFC. Cleavage of substrate was measured as the generation of fluorescence. Data were fitted using the mixed model of enzyme inhibition. The mean K_i_ of KVD900 is 3.02 nM ± 0.33 (mean ± SD; *n* = 3). The α of KVD900 was much greater than 1 indicating a competitive mode of inhibition. A representative graph is shown. K_i_, inhibitory constant; PKa, plasma kallikrein; α, alpha value

KVD900 competes with substrate as a reversible competitive inhibitor of human PKa with an estimated K_i_ of 3.02 nM ± 0.33 (mean ± SD; *n* = 3) (Figure 1). The IC_50_ of KVD900 for PKa, calculated from its K_i_, is 6.0 nM and its selectivity is >1000‐fold compared with the IC_50_ for a panel of human serine proteases, including FXIIa and FXIa (IC_50_ >40 µM) and tissue kallikrein (KLK1, IC_50_ >10 μM) (Table 1). The apparent association constant of KVD900 for PKa was above the limit of detection of 10 × 10^6^ M^−1^ s^−1^ (Table S1). The effects of KVD900 on PKa activity in whole control plasma (undiluted pooled human plasma) were evaluated in the presence of two concentrations of DXS. In the presence of 6.25 μg/ml DXS, PKa activity in plasma is 11.2 ± 1.0 fluorescence units/second (mean ± SD; *n* = 6) and the KVD900 IC_50_ is 54 ± 17 nM. A higher concentration of DXS (10 μg/ml) increased PKa activity in plasma to 13.8 ± 0.5 fluorescence units/second (mean ± SD), and the KVD900 IC_50_ to 74.1 ± 26 nM. Increased concentrations of isolated PKa are correlated with increased KVD900 IC_50_ (Figure S1).

**FIGURE 2:**
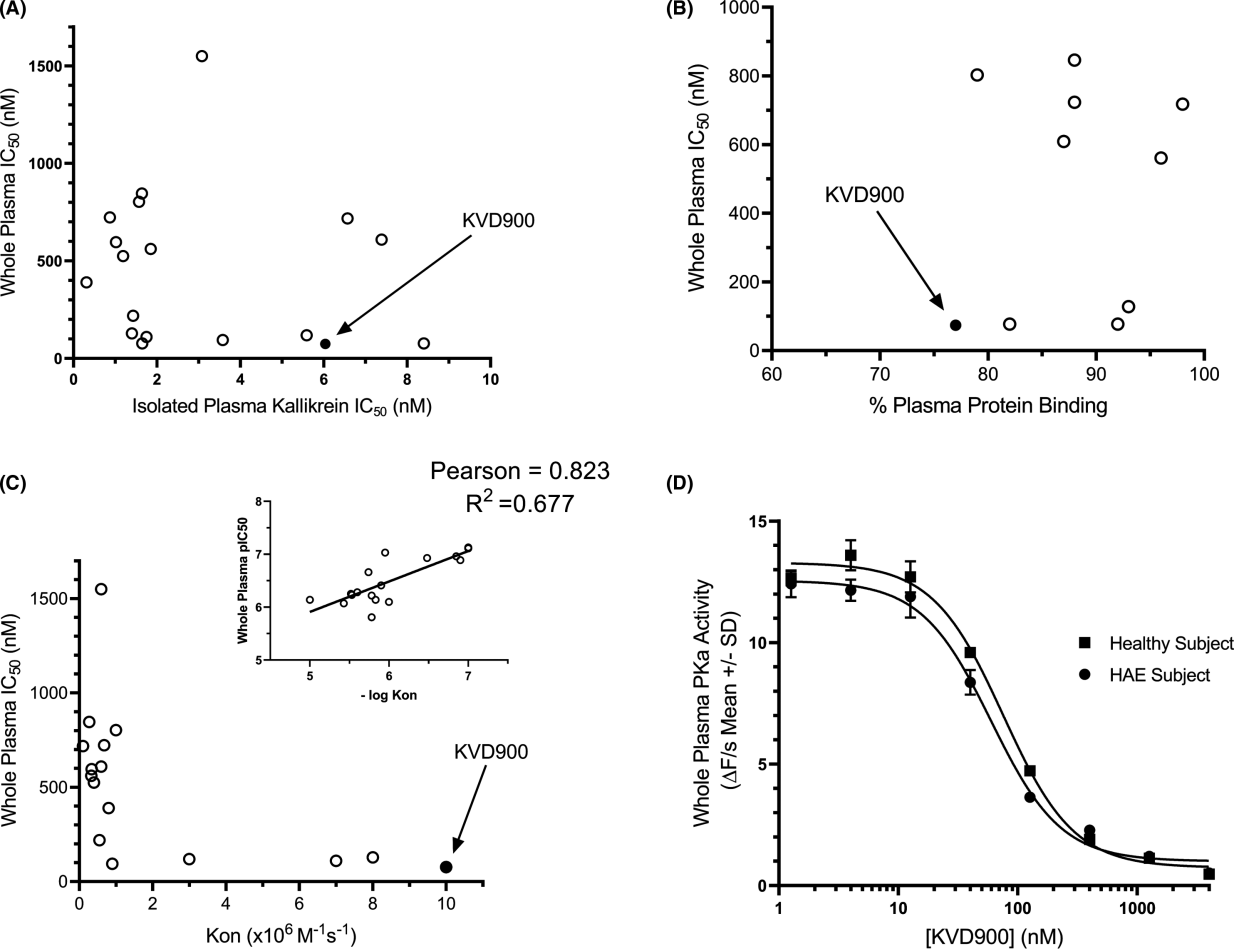
Comparison of the pharmacological properties of small‐molecule PKa inhibitors including KVD900. A panel of structurally diverse PKa inhibitors, including KVD900, with isolated enzyme IC_50_ <10 nM were tested in various assays to determine which properties influence potency in the DXS‐activated plasma assay. (A) PKa whole plasma potency is compared with PKa‐isolated enzyme potency, (B) PKa whole plasma potency is compared with percent plasma protein binding (%PPB) (C) PKa whole plasma potency is compared with K_on_ (a negative log transformation is used to highlight the correlation between these two variables. Compounds with a K_on_ >10 × 10^6^ M^−1^ s^−1^ are assigned a value of 10. (D) Representative graphs showing dose response effects of KVD900 in healthy control and HAE plasma. The data from KVD900 are shown in the solid symbols. DXS, dextran sulphate; IC_50_, half maximal inhibitory concentration; K_on_, association rate constant; PKa, plasma kallikrein; PPB, plasma protein binding

The IC_50_ for KVD900 in DXS‐stimulated plasma was among the lowest of a panel of other small molecule (<600 Dal) PKa inhibitors with IC_50_s <10 nM for isolated PKa (Table S1, Figure 2A). The IC_50_ of these PKa inhibitors for PKa in healthy control undiluted plasma stimulated with DXS did not correlate with their IC_50_ for isolated PKa enzyme (Figure 2A) or plasma protein binding (*R* ^2^ = .006) (Table S1, Figure 2B). Analysis of the rate constants of association (K_on_) for PKa revealed that the K_on_ for KVD900 was >10 × 10^6^ M^−1^ s^−1^. A negative correlation of whole plasma pIC_50_ and – Log (K_on_) was observed (Figure 2C, *R* ^2^ = .677). In contrast, the K_on_ for this panel of PKa inhibitors did not correlate with their isolated PKa IC_50_ (Table S1, Figure 2A, *R* ^2^ = .035). The IC_50_ of KVD900 added exogenously to individual pre‐dose HAE plasma samples followed by stimulation with 10 µg/ml DXS was 47.5 nM ± 10.4 (geometric mean ± SD, *n* = 6), which was not statistically different (*p* = .32) from the KVD900 IC_50_ measured in individual healthy subject plasma IC_50_ 54.4 nM ±13.1 (geometric mean ± SD, *n* = 6) (Figure 2D).

**FIGURE 3:**
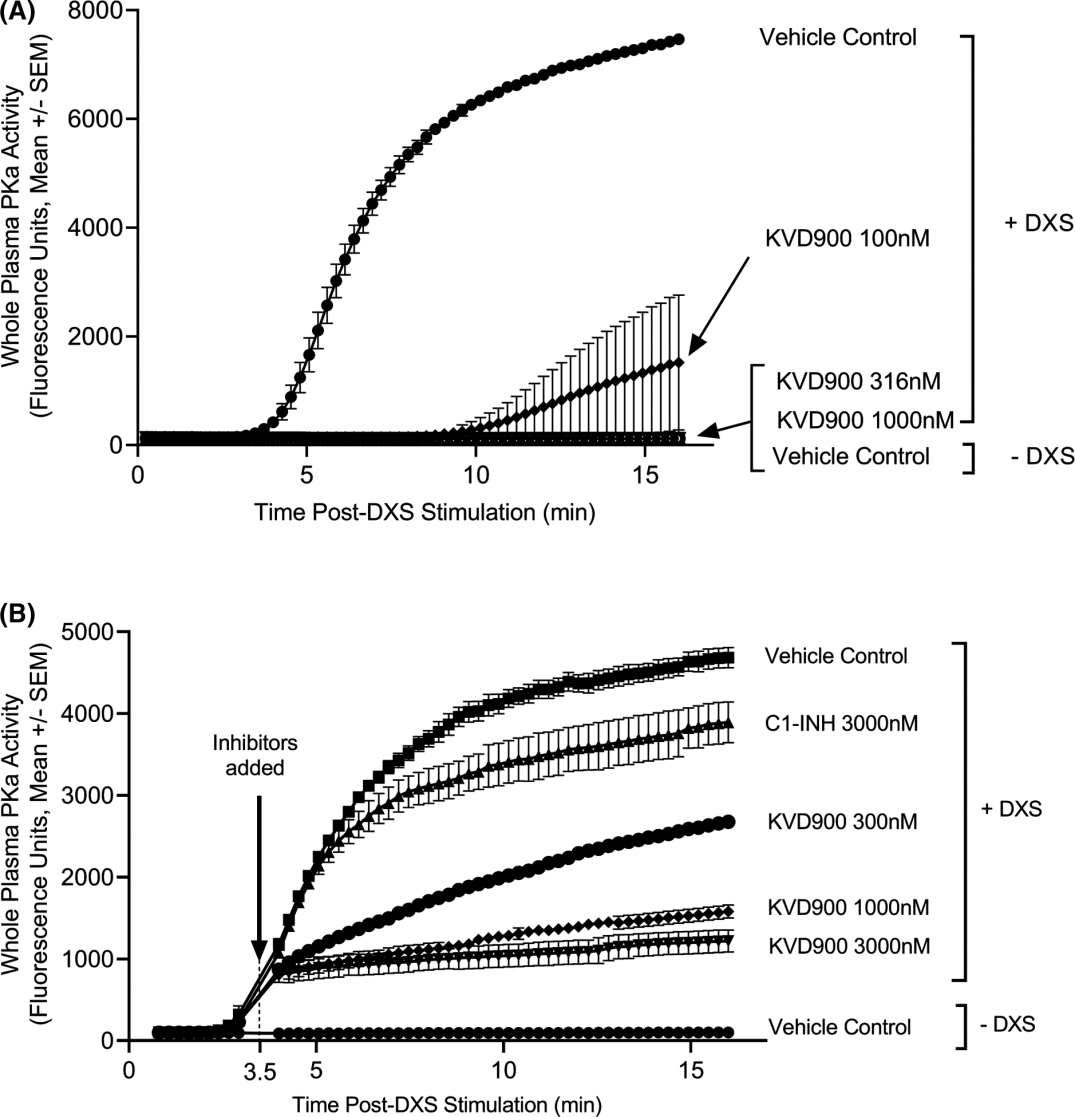
Effects of KVD900 and C1‐INH on PKa activity in human plasma. (A) Effects of KVD900 on PKa activity when undiluted plasma was pre‐treated with inhibitor for 5 min prior to stimulation of DXS (6.25 µg/ml). Average fluorescence units ± SEM from a single representative experiment are shown. (B) Comparison of the effects of KVD900 and C1‐INH on PKa activity in undiluted plasma pre‐stimulated with DXS (10 µg/ml). Inhibitors were added to plasma 3.5 min after the addition of DXS. The graph shows the average fluorescence units across a 16‐min assay in control human plasma (mean ± SEM, *n* = 3). Plasma was activated on ice and the catalytic assay was performed at 25°C. C1‐INH, C1 inhibitor; DXS, dextran sulphate; PKa, plasma kallikrein; SEM, standard error of the mean

The kinetic profile of DXS‐stimulated PKa activity in whole plasma displayed a lag of approximately 3 min before the appearance of the sigmoidal generation of fluorescence (Figure 3A). A 5 min pre‐incubation of plasma with 100 nM KVD900 prolonged this lag to approximately 9 min. Increasing the concentration of KVD900 to 316 and 1000 nM resulted in almost no detectable generation of fluorescence over the 17 min duration of the assay (>99.7% reduction compared with vehicle). The effects of KVD900 on pre‐activated control plasma were studied by the addition of KVD900 to plasma 3.5 min after DXS‐stimulation. Addition of 300, 1000 and 3000 nM KVD900 decreased the rate of fluorescence generation by 73.4%, 88.9% and 93.0% respectively (Figure 3B). KVD900 (300 nM) was more rapid and effective than 3000 nM (1.2 IU) of exogenously added C1‐INH in reducing DXS‐stimulated PKa activity in whole plasma (Figure 3B).

### 3.2 KVD900 protects HK from DXS‐stimulated cleavage

PKa‐mediated cleavage of HK generates bradykinin, which is difficult to accurately quantify due to its short half‐life in plasma.^19^ Changes in levels of HK and cleaved HK (cHK) are commonly used as biomarkers to assess PKa activity and bradykinin generation.^20^, ^21^ Capillary‐based immunoassays were used to create standard curves for KKS proteins and the *R* ^2^ correlations calculated using peak area chemiluminescence detected by immunoassays are HK *R* ^2^ = .95, PK *R* ^2^ = .95; FXII *R* ^2^ = .97, FXIIa *R* ^2^ = .81 (Figures [Link], [Link], [Link], [Link]). Using these standard curves we estimated the concentrations of HK, PK and FXII in whole plasma to be 92.1 ng/μl, 41.1 ng/μl and 22.5 ng/μl respectively.

**FIGURE 4:**
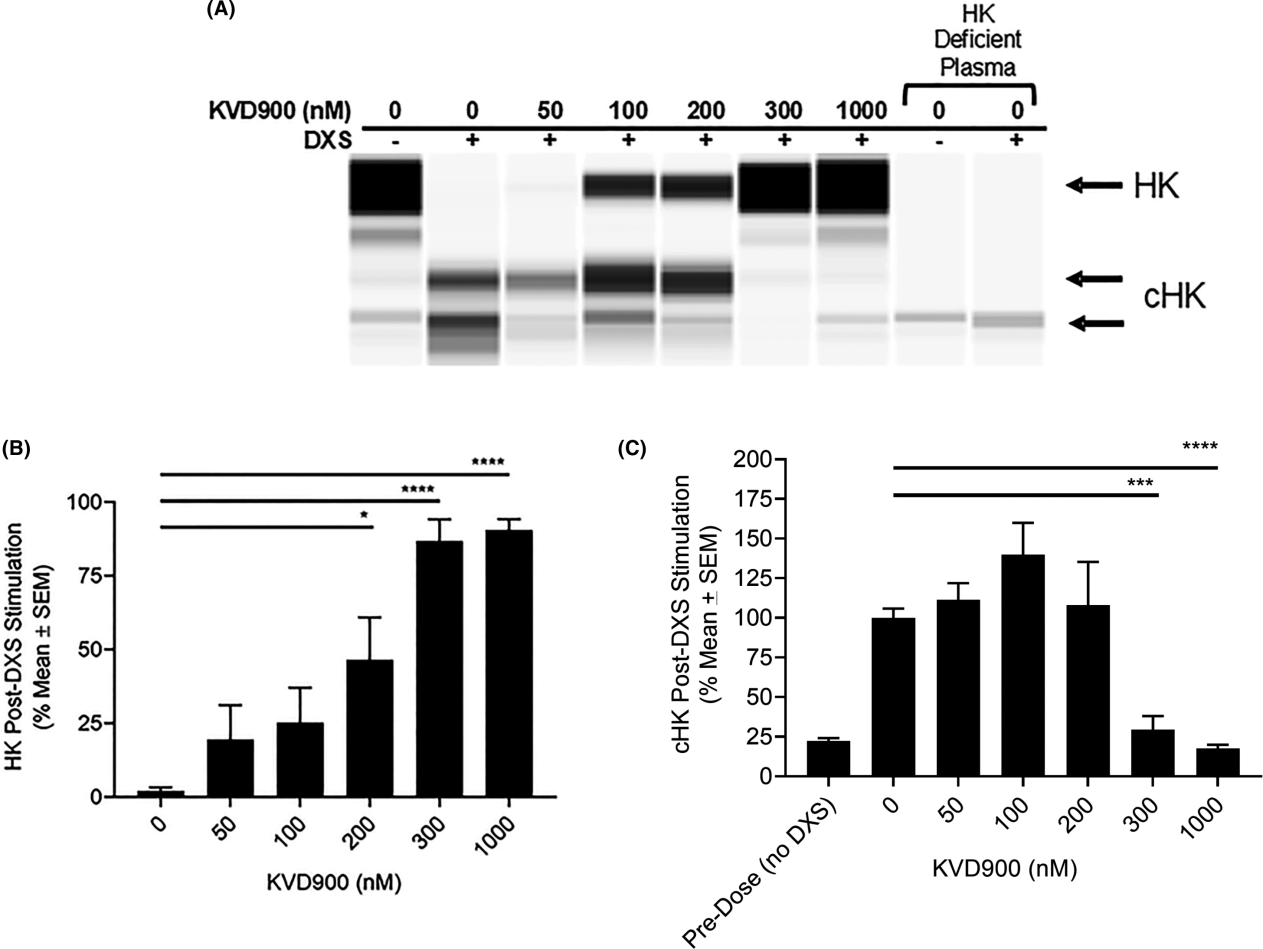
Effect of KVD900 on DXS‐stimulated HK cleavage in human plasma. The percent of HK and cHK post DXS‐stimulation (6.25 µg/ml) of the plasma on ice for 17 min were determined in the presence or absence of KVD900. A representative image generated from capillary electropherograms is shown (A). Bar graphs showing percent of HK (B) or cHK (C) post DXS‐stimulation (expressed as % mean ± SEM) from 5 independent experiments are plotted. *p*‐values of ≤.05 were considered statistically significant. cHK, cleaved HK; DXS, dextran sulphate; HK, high‐molecular‐weight kininogen; SEM, standard error of the mean. (*p*‐values: ****<.0001, ***<.001, *<.05)

The effects of KVD900 on DXS‐stimulated changes in HK and cHK were quantified in plasma. The specificity of HK and cHK detection was confirmed by the absence of immunoreactivity in HK‐depleted plasma. In the absence of KVD900, DXS‐stimulation decreased HK by 98%. The addition of KVD900 to plasma protected HK from DXS‐stimulated depletion in a concentration‐dependent manner with an IC_50_ of approximately 200 nM (Figure 4A,B). DXS‐stimulated generation of cHK in plasma was fully inhibited in the presence of 300 nM and 1000 nM KVD900 (Figure 4C).

### 3.3 Pharmacodynamic effects of orally administered KVD900 in first in human study

**FIGURE 5:**
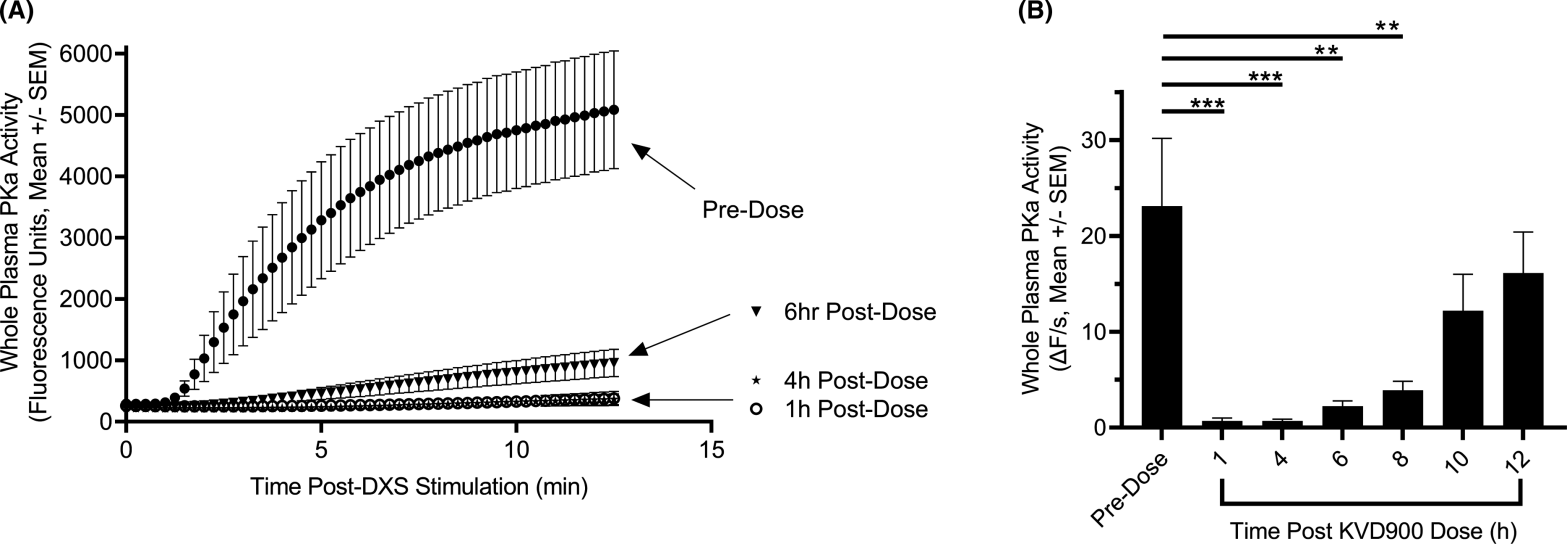
Effect of orally administered KVD900 on DXS‐stimulated PKa activity in healthy control plasma. (A) Catalytic activity of PKa in plasma samples from the KVD900 phase 1 trial was determined using a fluorogenic substrate after DXS‐stimulation (10 µg/ml). The graph illustrates the average fluorescence units of all subjects (*n* = 6) across a 12.5‐min assay. (B) The maximum rate of amidolytic activity at different time points post KVD900 600‐mg dose compared with pre‐dose. DXS, dextran sulphate; PKa, plasma kallikrein; SEM, standard error of the mean. Plasma was activated on ice and the catalytic assay was performed at 25°C. (*p*‐values: ***<.001, **<.01)

The effects of KVD900 on DXS‐stimulated PKa activity were evaluated in plasma obtained at pre‐dose and selected time points after oral administration of 600 mg KVD900, formulated as powder in capsule, in healthy male volunteers. DXS‐stimulated PKa activity in plasma samples was inhibited by >97% at 1 and 4 h (*p* < .001), >90% at 6 h (*p* = .0015) and >83% at 8 h post‐dose (*p* = .0024) compared with plasma obtained pre‐dose. (Figure 5).

**FIGURE 6:**
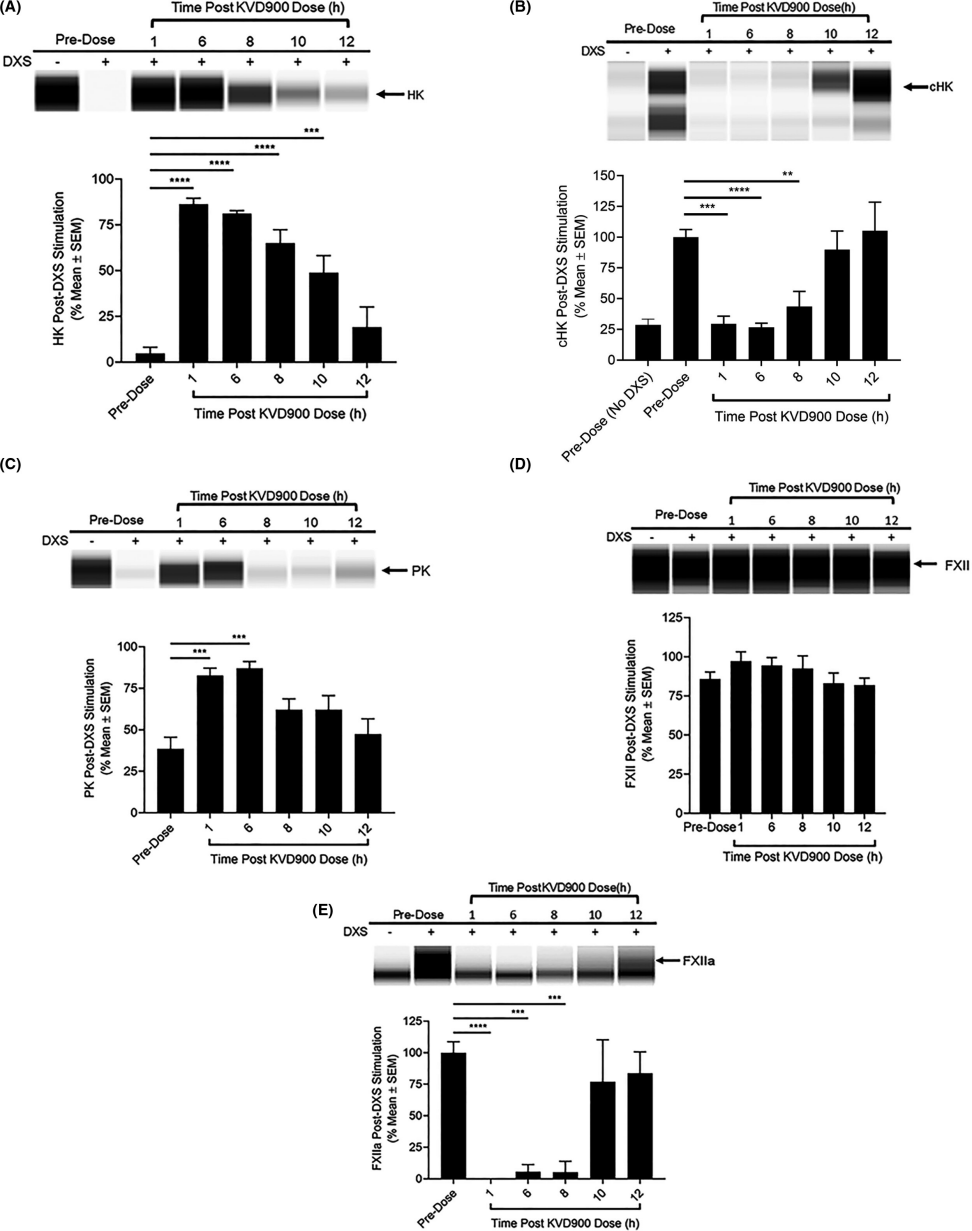
Effect of orally administered KVD900 on kallikrein kinin system proteins. Levels of kallikrein kinin system proteins were evaluated in a capillary‐based immunoassay in DXS‐stimulated (6.25 µg/ml) undiluted plasma (17 min on ice) from 6 healthy adult men after oral administration of 600 mg of KVD900. Representative images generated from capillary electropherograms are shown, along with corresponding bar graphs displaying mean percent ± SEM for (A) HK, (B) cHK, (C) PK, (D) FXII, and (E) FXIIa. cHK, cleaved HK; DXS, dextran sulphate; HK, high molecular weight kininogen; PK, plasma prekallikrein; SEM, standard error of the mean. (*p*‐values: ****<.0001, ***<.001, **<.01).

DXS‐stimulation of plasma samples obtained at pre‐dose resulted in near complete depletion of HK (4.7 ± 3.4% remaining compared with HK in unstimulated pre‐dose plasma, Figure 6A). Plasma samples obtained from 1 to 8 hours post‐dose with 600 mg KVD900 were protected from DXS‐stimulated HK cleavage (>65% HK remaining) compared with plasma obtained at pre‐dose (*p* < .0001). cHK levels in DXS‐stimulated plasma samples at 1, 6 and 8 h post‐dose were reduced compared with DXS‐stimulated pre‐dose plasma and were not significantly increased compared with pre‐dose without DXS (*p* ≥ .97, Figure 6B).

We examined the effects of KVD900 on the protection of PK and FXII in plasma samples from healthy subjects. DXS‐stimulation of pre‐dose samples decreased PK to 38.5 ± 7.0% of PK levels in unstimulated plasma (Figure 6C), suggesting that DXS activated 25.3 ng/μl of the 41.1 ng/µl PK contained in pre‐dose samples (Figure S3). In plasma samples obtained at 1 and 6 h post‐dose, DXS‐stimulation reduced PK to 82.7 ± 4.4% and 87.0± 4.1% of unstimulated plasma respectively. Plasma samples at 1 and 6 hours post dose were protected from DXS‐stimulated consumption of PK compared with pre‐dose plasma (*p* < .005). The DXS‐stimulated decrease in PK in plasma obtained at later time points (≥8 h) was comparable to the PK decrease observed in pre‐dose samples. A trend was observed for FXII protection in DXS‐stimulated plasma at 1 and 6 h post‐dose compared with pre‐dose plasma (non‐significant, Figure 6D). DXS‐stimulated generation of FXIIa was reduced by >90% in plasma obtained at 1, 6 and 8 h post‐dose with KVD900 compared with pre‐dose plasma (Figure 6E; *p* ≤ .0001 for 1, 6 and 8 h).

### 3.4 Pharmacodynamic effects of orally administered KVD900 in HAE

**FIGURE 7:**
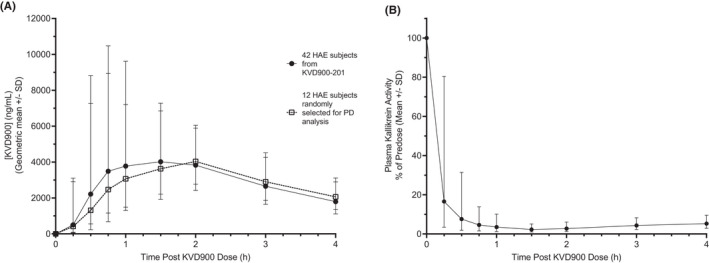
Effect of orally administered KVD900 on DXS‐stimulated PKa activity in HAE plasma. (A) Plasma concentrations of KVD900 following a single 600 mg oral administrations in tablet formulation to HAE subjects. Solid line is the geometric mean ± standard deviation for full *n* = 42 subjects enrolled and dashed line indicated mean concentrations from the 12 subjects used for PKa activity measurements. (B) Catalytic activity of PKa in plasma samples from the KVD900 phase 2 trial was determined using a fluorogenic substrate after DXS stimulation (10 µg/ml). The maximum rate of amidolytic activity at different time points post KVD900 600‐mg dose compared with pre‐dose. DXS, dextran sulphate; PKa, plasma kallikrein; SD, standard deviation. (*p*‐values: ***<.001, **<.01).

**FIGURE 8:**
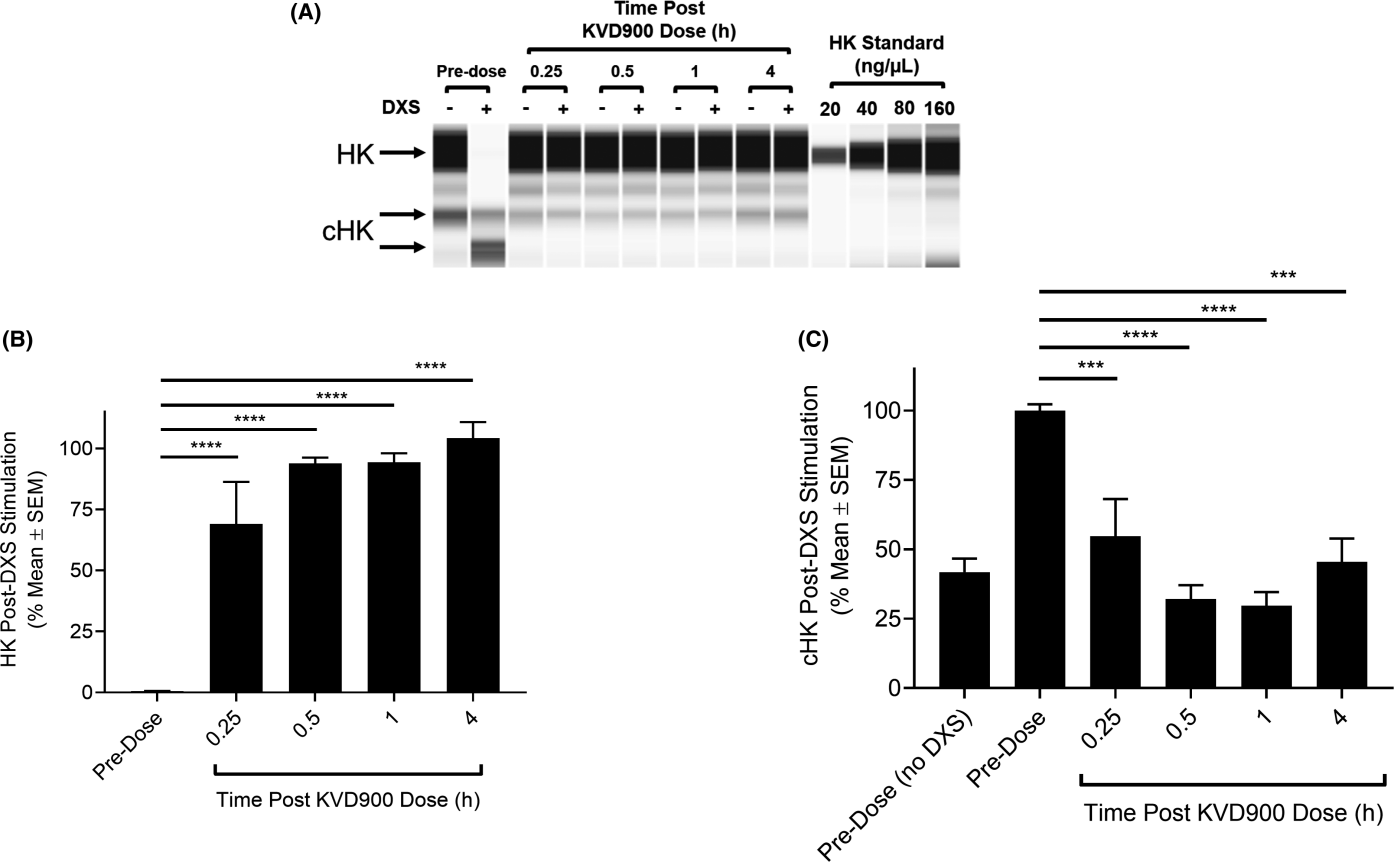
Effect of orally administered KVD900 on DXS‐stimulated PKa activity in HAE plasma. The percent of HK and cHK in plasma post stimulation with 6.25 µg/ml on ice were determined at pre‐dose and at the times indicated up to 4 h post‐dose. An image generated from capillary electropherograms is shown (A). Bar graphs showing percent of HK (B) or cHK (C) post DXS‐stimulation (% mean ± SEM) from 6 subjects with HAE are plotted. *p‐*values of ≤.05 were considered statistically significant. cHK, cleaved HK; DXS, dextran sulphate; HK, high molecular weight kininogen; SEM, standard error of the mean. (*p*‐values: ****<.0001, ***<.001)

Orally administered KVD900 (600 mg), formulated in tablets, was rapidly absorbed in HAE Type 1 and Type 2 subjects with mean 6570 ng/ml C_max_ and 1.2 h T_max_ (arithmetic mean, *n* = 42) (Figure 7A). A randomly selected subset of 12 patients from the total cohort of 42 were chosen for pharmacodynamic analysis of PKa activity in whole plasma. The mean pharmacokinetic profile of KVD900 in this subset of 12 patients was similar to the full 42 cohort (Figure 7A). DXS‐stimulated mean PKa activity in plasma samples from HAE subjects was inhibited by ≥95% at 45 min post oral dosing and this level of near complete inhibition of PKa was maintained for at least 4 h post‐dose (Figure 7B). Plasma samples from six HAE patients that received KVD900 were further analysed using the DXS‐stimulated HK cleavage assay. Using a standard curve with purified HK, we estimated that the concentration of HK in HAE pre‐dose plasma in this cohort was 76.0 ± 26.1 ng/μl (Figure 8A). In pre‐dose samples, DXS‐stimulation induced near complete depletion of HK with corresponding increase in the appearance of cHK (Figure 8A,B). DXS‐stimulated plasma samples obtained 15 min through 4 h post‐dose were strongly protected from DXS‐stimulated depletion of HK and displayed cHK levels comparable with unstimulated plasma (Figure 8A,B).

## 4 DISCUSSION

KVD900 was identified as a potent, competitive, reversible and selective PKa inhibitor with fast association (K_on_) kinetics. We show that orally administered KVD900 provides rapid and near complete inhibition of DXS‐stimulated PKa activity and HK cleavage in plasma from both healthy controls and individuals with HAE. Moreover, we show that KVD900 suppressed KKS activation, which may contribute to the generation of PKa during attacks.

Currently available on‐demand treatments for HAE attacks are parenterally administered inhibitors of PKa,^22^ an antagonist of the B2R^23^ or replacements of C1‐INH.^24^, ^25^ We sought an orally administered PKa inhibitor that provides both fast and highly effective suppression of HK cleavage and KKS activation. The effects of KVD900 on PKa enzyme activity and KKS activation in a whole plasma assay system were characterized using DXS, which causes a rapid increase in PKa enzyme activity and depletion of HK. This model of HK depletion was intended to mimic the KKS activation that may occur locally in affected tissue during a HAE attack. We used the model to examine the effects of KVD900 on PKa activity, HK cleavage and positive feedback activation of the KKS on PK and FXII in plasma samples obtained from healthy volunteers in a phase 1 trial and HAE patients from a phase 2 trial.

### 4.1 Pharmacological characterization of KVD900

We demonstrated that PKa inhibitor IC_50_ for isolated PKa did not correlate with PKa inhibition IC_50_ in whole plasma, suggesting that other properties beyond the IC_50_ for isolated PKa might affect potency of PKa inhibition in DXS‐stimulated whole plasma. Using a standard curve with purified PK, the concentration of PK (86 kDa) in normal plasma was estimated at 41.1 ng/µl (478 nM), which is comparable to the estimated range of 35–50 ng/µl (407 to 581 nM) previously reported.^26^ DXS‐stimulation of plasma resulted in depletion of HK (Figure S3 and Figure 6A), and an approximate 60% decrease in PK (Figure 6C) corresponding to the potential generation of up to 24.7 ng/µl (287 nM) PKa. These changes in PK and PKa concentrations in the DXS‐stimulated whole plasma assay exceed the estimated 75–186 nM decrease in PK, and the 20–100 nM increase in PKa measured during an untreated HAE attack.^27^, ^28^, ^29^, ^30^ Since PKa activity in unstimulated plasma is low (Figure 3A), our whole plasma assay appears to model the range of KKS activation from quiescence in unstimulated control plasma to PKa concentrations that have been reported to occur during an HAE attack. Using a panel of potent PKa inhibitors (isolated PKa IC_50_ <10 nM), we show that isolated and whole plasma PKa potency do not correlate (Figure 2B). A number of factors could affect the pharmacology of PKa inhibitors in plasma, including plasma protein binding and the association kinetics K_on_ for PKa. Plasma protein binding alone is not sufficient to explain potency in plasma since KVD900 and KV998063 have similar PPB, 77% and 79% respectively, and the IC_50_ of KVD900 is 10‐fold lower than KV998063 in plasma (Table S1). Interestingly, we found that the K_on_ of >10 × 10^6^ M^−1^ s^−1^ for KVD900 was among the highest in this series of inhibitors. K_on_ did not correlate with the IC_50_ for isolated PKa (*R* ^2^ = .035; Pearson correlation = −0.188, data not shown) but did so significantly with whole plasma IC_50_ (*R* ^2^ = .677; Pearson correlation = .823) (Figure 2C). The K_on_ of KVD900 is also faster than the reported 3.4 × 10^6^ M^−1^ s^−1^ for the PKa inhibitor DX‐2930 (lanadelumab) and orders of magnitude faster than the 1.7 × 10^4^ M^−1^ s^−1^ estimate for C1‐INH.^31^ We conclude that the fast association kinetics (K_on_) of KVD900 for PKa contributes to its low IC_50_ in plasma.

Increased concentrations of isolated PKa are correlated with increased KVD900 IC_50_ (Figure S1) and as a result the higher IC_50_ in the whole plasma assay compared with the IC_50_ in the isolated PKa assay is an expected observation, as the amount of PKa generated following DXS‐stimulation of plasma is considerably more than the 0.1 nM PKa used in the isolated enzyme PKa assay.^32^ The increased IC_50_ for KVD900 in plasma stimulated with 10 µg/ml compared with 6.25 µg/ml DXS is consistent with a higher amount of PKa enzyme activity generation with the higher DXS concentration. As the PKa enzyme concentration in the assay influences the observed IC_50_, it should be considered when evaluating potency in plasma‐based assays for PKa. Reducing the enzyme concentration by dilution of the plasma has the expected effect of lowering the IC_50_ of the compound; KVD900 IC_50_ in DXS‐activated 1:4 diluted plasma is 10.4 nM (data not shown). The IC_50_s of KVD900 in healthy control and HAE plasma using the undiluted whole plasma PKa assay were similar to each other, which suggests utility for this assay in translating results between phase 1 studies in healthy volunteers and phase 2 studies in HAE patients.

### 4.2 Effects of orally administered KVD900 on PKa enzyme activity, HK cleavage, and KKS activation

We demonstrate oral administration of 600 mg KVD900 leads to a >97% inhibition of DXS‐stimulated PKa activity in plasma samples from healthy volunteers at 1 and 4 h post‐dose and that prolonged stimulation with DXS did not overcome PKa inhibition by KVD900. The fast inhibitory effects of oral KVD900 on the whole plasma PKa activity obtained following administration of powder in capsule formulation through 8 h post dose are comparable with results from KVD900 administered in tablet formulation.^33^ HAE attacks are associated with increased concentrations of cHK in plasma.^21^ Banerji *et al*.^5^ demonstrated that clinical efficacy of lanadelumab in reducing HAE attacks is associated with a reduction in circulating cHK and a decrease in cHK generated in plasma‐stimulated *ex vivo* with FXIIa. We showed that KVD900 rapidly provided protection from DXS‐stimulated HK cleavage from 15 min post‐dose and for at least 10 h after oral administration. Moreover, DXS‐stimulated generation of cHK were not detectable for at least 6 h.

We show that KVD900 protects against DXS‐induced consumption of PK and the generation of FXIIa at 1 and 6 h post‐dose (Figure ). The small decrease in PK that occurs in the presence of strong PKa inhibition could be due to a low level of remaining KKS activation and/or FXII‐mediated activation of PK that may occur without KKS feedback activation.^34^ The inhibitory effect of KVD900 on the cleavage of PK is consistent with the role of PKa in the feedback activation of FXII by the KKS, and protection of HK, PK, and FXII from DXS‐stimulated cleavage (Figure 6). These results suggest that KVD900‐mediated suppression of both PKa and FXIIa generation during KKS activation may reduce both HK cleavage and the propagation and spread of KKS activation during an HAE attack. Suppressing the generation of PKa and FXIIa during an HAE attack would further reduce the uncontrolled effects of the KKS that are independent of BK action, including the potential effects on complement and coagulation cascades.^35^, ^36^

We show that the effects of orally administered KVD900 on DXS‐stimulated PKa activity and HK cleavage observed in HAE subjects enrolled in the phase 2 trial were consistent with the observations of the pharmacodynamic effects of KVD900 in healthy volunteers. Both studies demonstrated that KVD900 provided near complete inhibition of PKa activity. In the HAE patients, we showed that inhibition of PKa activity and HK cleavage are observed from 15 min post‐dose, demonstrating a rapid PD effect following oral administration of KVD900.

In conclusion, KVD900 is an orally available PKa inhibitor that has been selected as a candidate for on‐demand therapy for HAE, in part, for its high potency in whole plasma and rapid and near complete inhibition of PKa following oral administration. In addition to strongly inhibiting HK cleavage, which would be expected to markedly reduce the production of BK, our pharmacological data suggest that KVD900 also inhibits PKa and FXIIa generation during KKS activation. Importantly, KVD900 can both prevent KKS activation and inhibit PKa post‐KKS activation. While early administration of on‐demand therapies is recommended, these properties are consistent with beneficial clinical effect if KVD900 is administered before or after the start of an attack. The combination of oral administration, rapid absorption, and near complete PKa inhibition and suppression of KKS activation is well suited to on‐demand treatment of HAE attacks. KVD900 provides an opportunity to rapidly halt cleavage of HK by PKa that leads to BK‐mediated oedema and suppress the further generation of PKa and FXIIa by the KKS, which may contribute to attack duration and severity.

## CONFLICT OF INTEREST

EJD, NM, LJR, DKL, CMY, SLH, and EPF are employees of KalVista Pharmaceuticals. LL, GMDD, and AM were employees of KalVista Pharmaceuticals at the time of the study.

## AUTHOR CONTRIBUTIONS

EJD and NM contributed equally to the experimental work, analysed data and edited the manuscript. LL, LJR, DKL and GMDD were involved in the experimental work, data collection and manuscript review. AM, CMY and SLH were involved in conceptualization, project administration, review and editing. EPF was involved in project conceptualization and design and wrote the manuscript. All authors provided critical input to the project and approved the final manuscript.

## Supporting information

Fig S1

Fig S2

Fig S3

Fig S4

Fig S5

Table S1

## Data Availability

The data that support the findings of this study are available from the corresponding author upon reasonable request.
