# Supplementary figures and images for: Pharmacological suppression of the kallikrein kinin system with KVD900: An orally available plasma kallikrein inhibitor for the on‐demand treatment of hereditary angioedema
Source: Clin Exp Allergy. 2022 Mar 20;52(9):1059–70. doi: 10.1111/cea.14122 (PMC9544254; doi:10.1111/cea.14122)

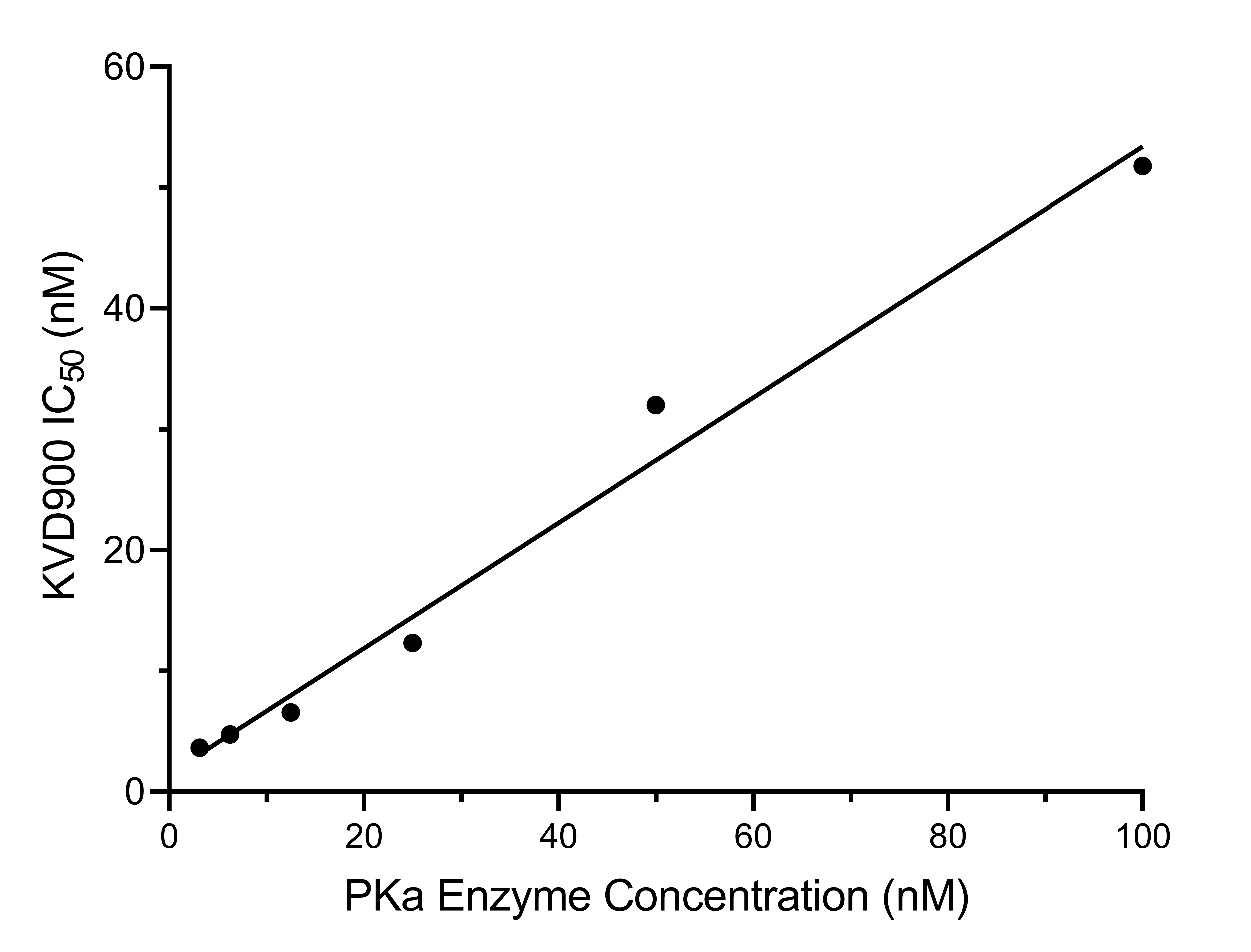

Supplement: Supplementary file 1 — Fig S1 [file CEA-52-1059-s001.tif]

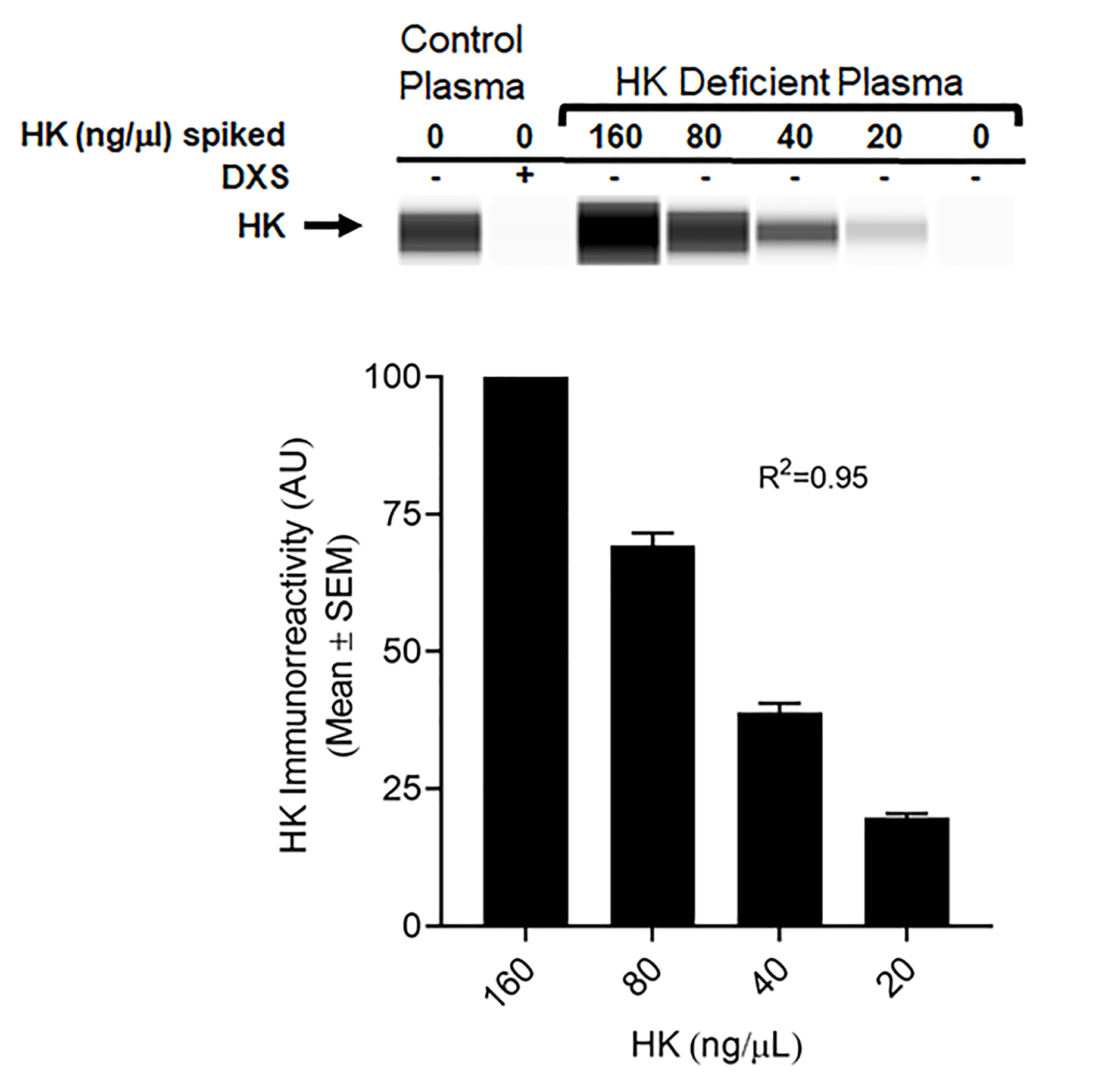

Supplement: Supplementary file 2 — Fig S2 [file CEA-52-1059-s003.tif]

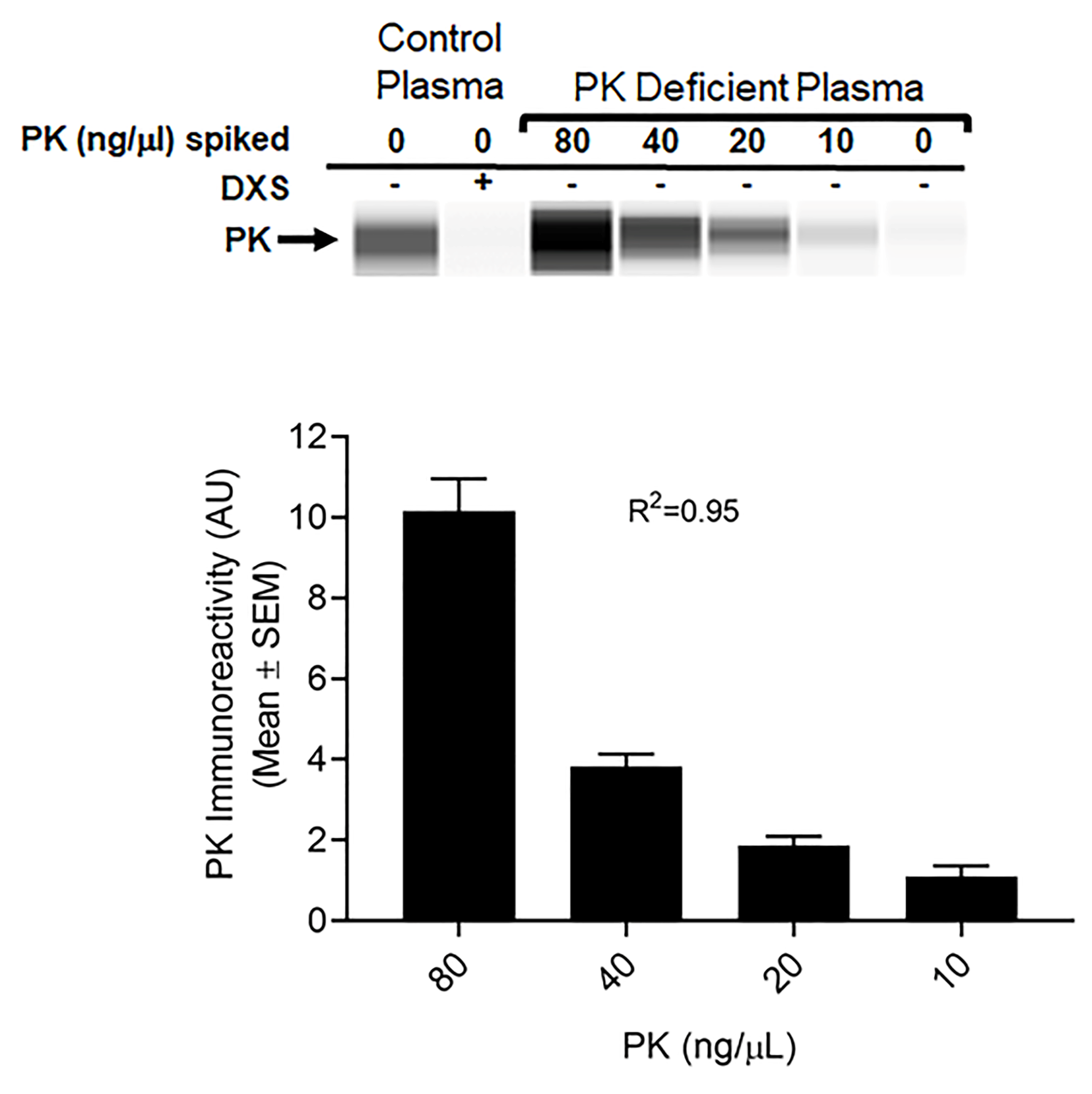

Supplement: Supplementary file 3 — Fig S3 [file CEA-52-1059-s006.tif]

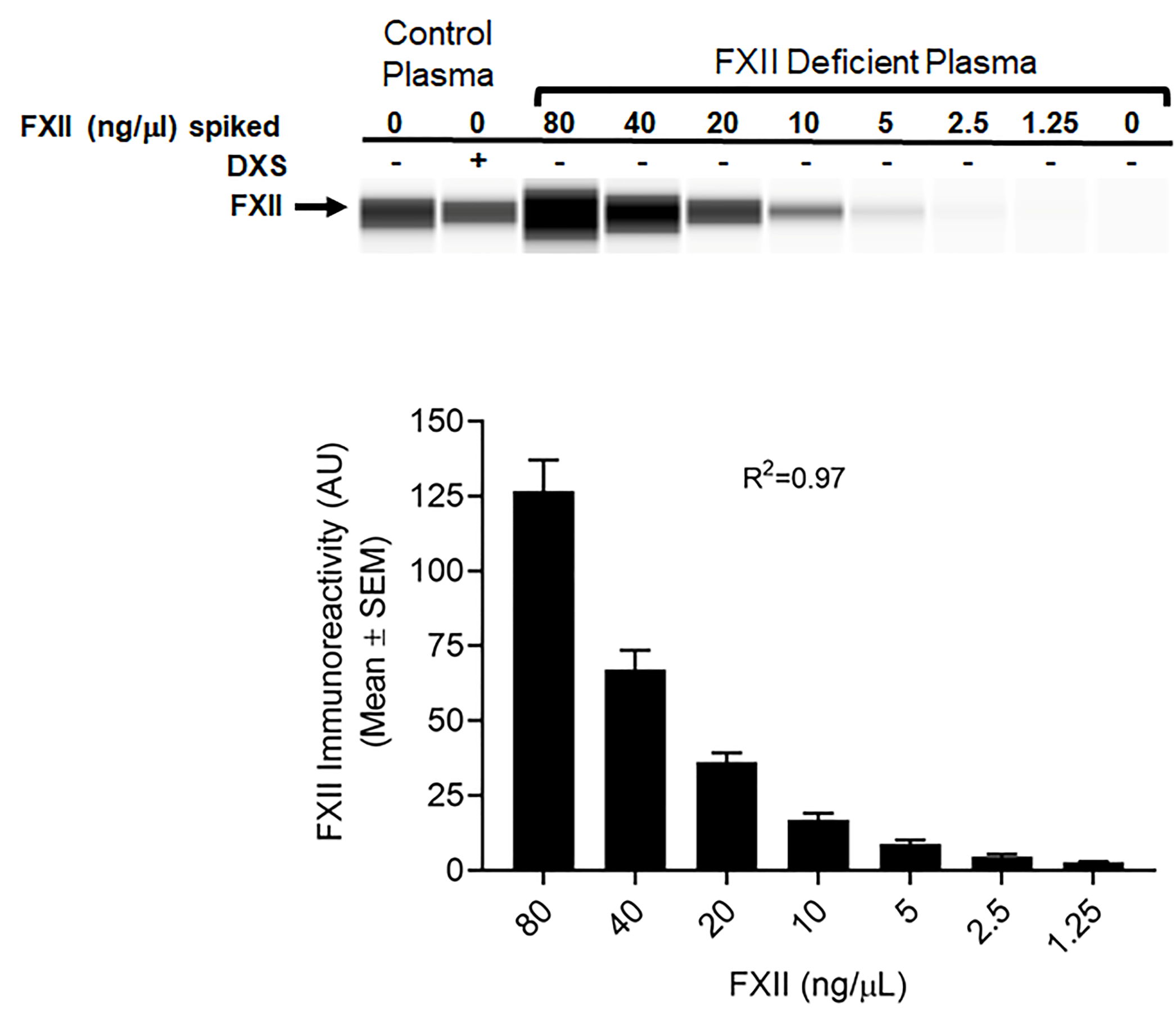

Supplement: Supplementary file 4 — Fig S4 [file CEA-52-1059-s004.tif]

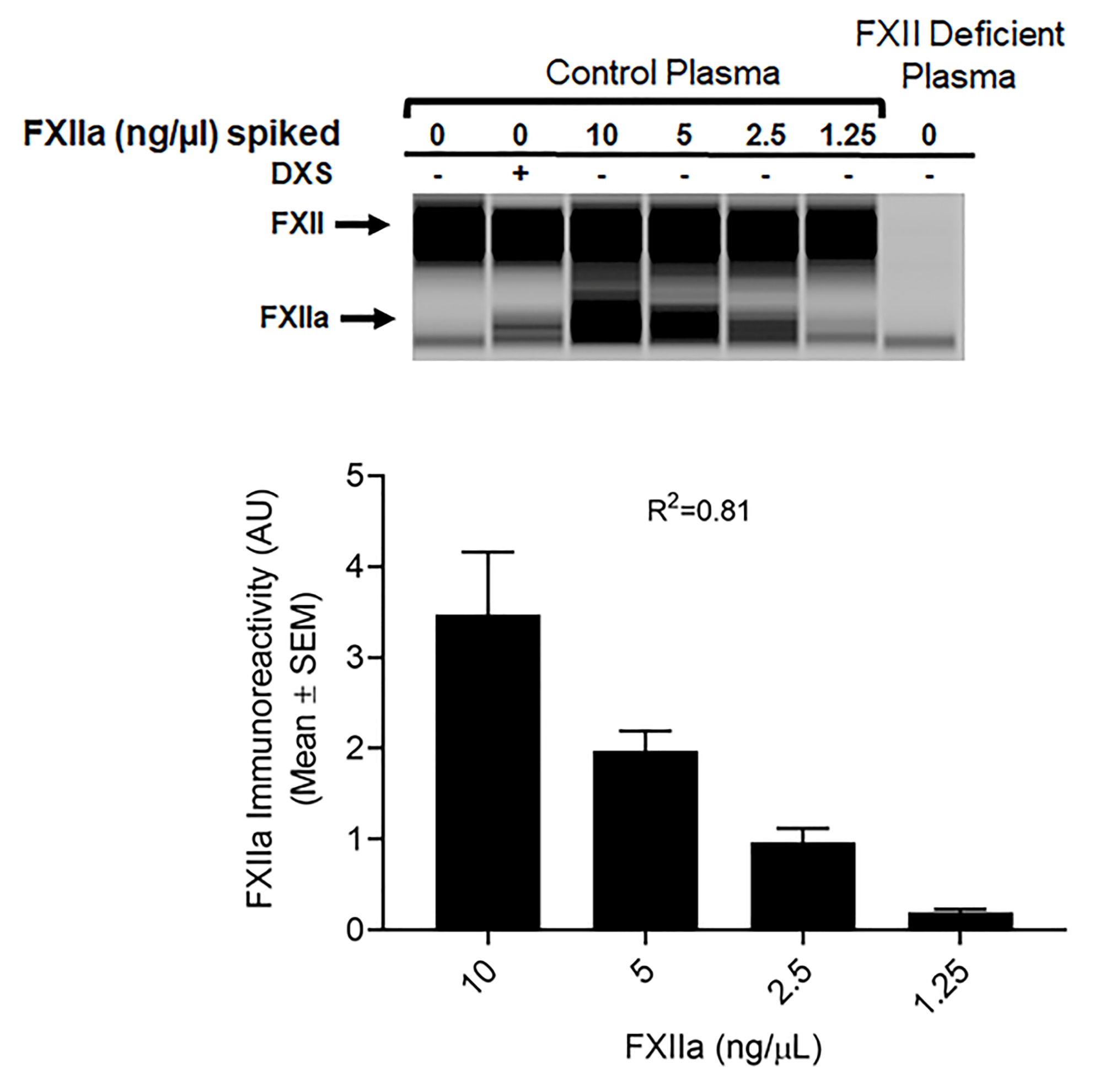

Supplement: Supplementary file 5 — Fig S5 [file CEA-52-1059-s005.tif]

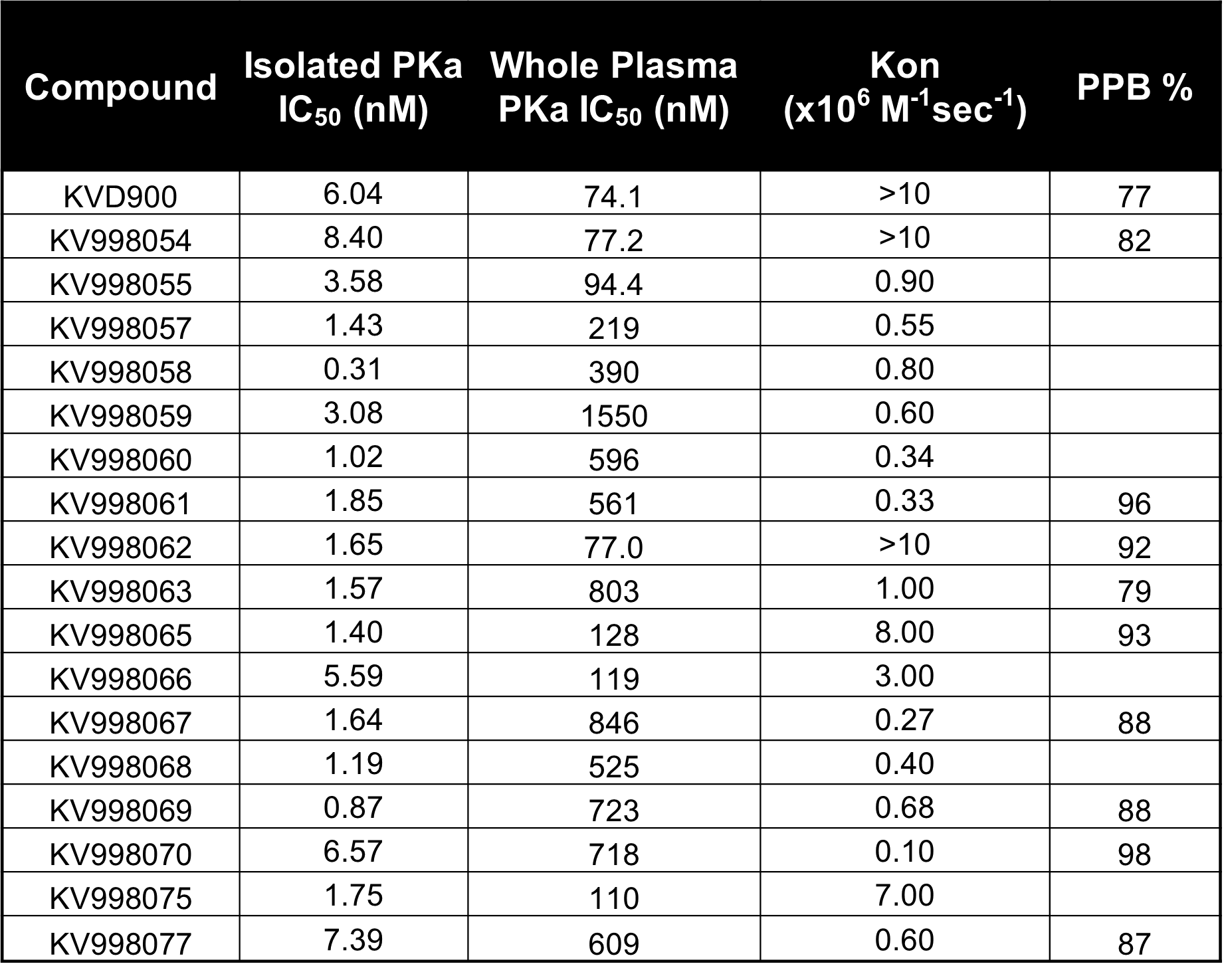

Supplement: Supplementary file 6 — Table S1 [file CEA-52-1059-s002.tif]
